# Effects of Educative Materials on Doctors' Intention to Initiate Life-Saving Procedures After a Suicide Attempt: Randomised Controlled Trial

**DOI:** 10.3389/fpsyg.2021.718084

**Published:** 2021-08-03

**Authors:** Marlies Braun, Thomas Niederkrotenthaler

**Affiliations:** Unit Suicide Research & Mental Health Promotion, Department of Social and Preventive Medicine, Center for Public Health, Medical University of Vienna, Vienna, Austria

**Keywords:** suicide, suicide prevention, assisted suicide, emergency medicine, educative media material, media effects, end-of-life decision

## Abstract

**Introduction:** The topic of euthanasia, assisted dying, and how to deal with death wishes has received strong public and media attention in many countries. Nevertheless, there is currently no research which has analysed if educative materials that favour or disfavour the initiation of life-saving measures after a suicide attempt impact on attitudes to initiate such procedures among physicians.

**Materials and Methods:** A double-blind randomised controlled trial was conducted to test if educative materials that either support life-saving measures or rather recommend against it after a near-fatal suicide attempt has an effect on intentions to initiate such measures (trial registration: DRKS00024953, www.drks.de). *N* = 192 doctors from the Medical University Vienna (Austria) participated in the study and either read educative materials not recommending (*n* = 59), or recommending life-saving measures (*n* = 64), or were not reading educative materials (*n* = 69, control group). The primary outcome was intentions to initiate life-saving measures in an open case vignette featuring the case of a terminally ill cancer patient. Other variables assessed were demographics, experiences with terminally ill and dying patients, training or qualification in mental health, specialty, position, whether doctors worked in emergency medicine, and attitudes toward assisted dying. A logistic regression analysis was used.

**Results:** There was no immediate effect of educative materials on intentions to initiate life-saving measures, χ^2^(2) = 0.94, *p* = 0.63. The adjusted model including all tested predictors was significant [χ^2^(15) = 37.82, *df* = 15, *p* < 0.001]. Attending position, male gender, low age, and more negative attitudes to assisted dying predicted a decision for life-saving measures. Higher agreement with life-saving measures was reported for a case vignette about a patient with schizophrenia than for a case vignette about a patient with Huntington's disease.

**Discussion:** Educative materials either favouring or disfavouring the initiation of life-saving measures after a suicide attempt do not appear to immediately influence related decision-making processes. Related intentions appear mainly influenced by personal opinions on the topic and by the specific patient case. Good-quality in-depth discussions regarding end-of-life decisions and to develop well-founded and non-opinionated guidelines are highly warranted.

## Introduction

Euthanasia and assisted dying are timely yet under-researched topic areas. The public debate over end-of-life decisions has become increasingly widespread around the world in recent years (Ryynänen et al., 2002; Emanuel et al., 2016). This is related to the broader discussion on how to tackle this issue in society, policy, and healthcare (Emanuel, 1994; Moskop and Iserson, 2001; Goligher et al., 2017), and an increasing number of countries have adopted increasingly liberal legislation in recent years (Emanuel et al., 2016). In the context of societal discussions about this topic, some research has assessed attitudes toward assisted dying and euthanasia (Fekete et al., 2002; Seale, 2009; Humphrey, 2011; McCarthy, 2014; Stolz et al., 2015). These studies have often investigated attitudes in the general public (Seale, 2009; Humphrey, 2011; McCarthy, 2014; Stolz et al., 2015; Yun et al., 2018), and several findings indicate that increasingly permissive attitudes have been adopted in the general public in many countries over time (Emanuel et al., 2016). A few studies, however, have also been conducted among healthcare staff (Fried et al., 1993; Fekete et al., 2002; Ryynänen et al., 2002; Seale, 2009; Yun et al., 2018; Abohaimed et al., 2019). Healthcare personnel, particularly physicians, are of high relevance in public discourse on the topic area, because these groups are typically directly involved in end-of-life decisions, and medical professions consistently enjoy a large authority in public perceptions, which is typically higher than that of other stakeholders including politicians (Fowid, 2018; Schönherr and Zandonella, 2020). Existing literature suggests that attitudes of doctors toward end-of-life-related attitudes are not well-understood. With regard to variables potentially underlying perspectives and opinions, more experience with palliative medicine and dying patients, appears to be associated with more negative attitudes toward assisted suicide (Grassi et al., 1999; Peretti-Watel et al., 2005; Abohaimed et al., 2019). Accordingly, some studies have found that specialties and disciplines frequently treating terminally ill patients, such as oncologists (Peretti-Watel et al., 2005) and palliative medicine specialists (Seale, 2009), were less approving of euthanasia and related topics. Further, some studies reported attitudes in favour of euthanasia and assisted dying more pronounced in male doctors (Seale, 2009; Abohaimed et al., 2019), older physicians (Peretti-Watel et al., 2005; Seale, 2009), and physicians in a lower position (Abohaimed et al., 2019). Patients' diagnosis and prognosis was also found to be influencing physicians' opinions about euthanasia and assisted dying, with a more accepting attitude in terminally ill patients (Ryynänen et al., 2002).

In the wake of legislation reforms such as those seen recently in many countries surrounding the topic of euthanasia and assisted suicide, media have always been relevant to form public opinion and clearly influences public debates (Jaye et al., 2021). Media reporting on the topic area is often highly opinionated, in accordance with strong attitudes pro or against assisted suicide in specific population groups (Rietjens et al., 2013). It remains unknown, however, if opinionated media items can actually impact attitudes toward the topic of assisted suicide. From the field of media and suicide research, there is some but very little evidence which suggests opinionated reporting might indeed have some immediate impact on public attitudes. Arendt and colleagues tested in a laboratory experiment if the specific choice of wordings in a media article might influence attitudes to suicide in a lay audience (Arendt et al., 2018). They found that participants reading a media item using more permissive language related to suicide showed more positive attitudes and greater support for suicide among individuals suffering from incurable diseases than participants who read the same educative media article, but with less permissive wording for suicide (Arendt et al., 2018). Accordingly, the way of presenting suicide related content in media appears to influence how individuals interpret and value suicide in the general public.

With regard to media effects in the area of euthanasia and assisted dying, there is even less research available. Particularly, there is a clear research gap related to the question if opinionated media articles about end-of-life decisions can influence professional decision-making among healthcare staff.

To bridge this research gap, we conducted a randomised controlled trial to investigate immediate media effects of opinionated educative materials in medical doctors. We tested written educative material either favouring the acceptance of a death wish raised by suicidal patients or disfavouring the acceptance in doctors and assessed any impact of the media item on their intentions to initiate life-saving measures.

Specifically, we tested the following hypotheses:

- Doctors presented with educative materials favouring the initiation of life-saving measures will report higher intentions to initiate life-saving measures after reading the considerations, whereas those reading the materials disfavouring the initiation of life-saving measures will be more opposed to initiate life-saving measures, as compared to the control group.
- Doctors reading educative materials favouring the initiation of life-saving measures will show stronger agreement with life-saving measures whereas those reading the materials disfavouring the initiation of life-saving measures will show stronger disagreement with life-saving measures, as compared to the control group, in different patients' scenarios.
- Doctors with more pro-assisted dying attitudes will be more opposed to initiate life-saving measures.

We also explored differences between gender, seniority level, specialty, and training.

## Materials and Methods

### Participants

For this study, medical doctors in clinical practise, with an Austrian citizenship or permanent residence in Austria, and good German skills were included to participate. We recruited doctors at the Medical University of Vienna. The Medical University and its university hospital, AKH, is one of the largest hospitals in Europe, with around 5.500 employees. Doctors were invited to participate in a 10-min online study via e-mail invitation, which disclosed that the study would test the impact of information material on medical decision-making in emergency situations. The announcements also stated that the material would bring up topics such as suicide or a patient's wish to die, without giving specific details.

### Randomisation, Blinding, and Allocation Concealment

We conducted an online, double-blind randomised controlled trial with two intervention groups and one control group from May to June 2020 (details on the study group conditions are provided in the section Materials and Procedure). All doctors of the Medical University of Vienna (*n* = 1.804 on reference date: 1 March 2021) were randomised to one of the three conditions before sending out individual e-mail invitations. Condition 1 included the reading of educative material disfavouring the initiation of life-saving measures; condition 2 included the same material but favouring life-saving measures, and condition 3 was the control group which did not receive any opinionated text (see below, procedure for more details). For randomisation, the human resources department of the Medical University of Vienna created an anonymized list of all doctors of the Medical University of Vienna using anonymized IDs (i.e., the numbers from 1 to 1.804). One of the researchers (MB) randomly assigned each anonymized ID to the numbers 1, 2, or 3, using the List Randomizer option at the www.random.org/lists website. We provided the human resources staff with the randomised list (i.e., a list of the numbers from 1 to 1.804 assigned to the numbers 1–3). Staff matched the anonymized IDs to doctors' names and e-mail addresses and sent out invitations to the online study consistent with the respective study arm. Three different study invitations were sent out with doctors assigned a 1 receiving a link to the study in experimental arm 1, doctors assigned a two receiving a link to the study in experimental arm 2, and doctors assigned a 3 receiving a link to the study in the control arm.

### Materials and Procedure

**Figure 1:**
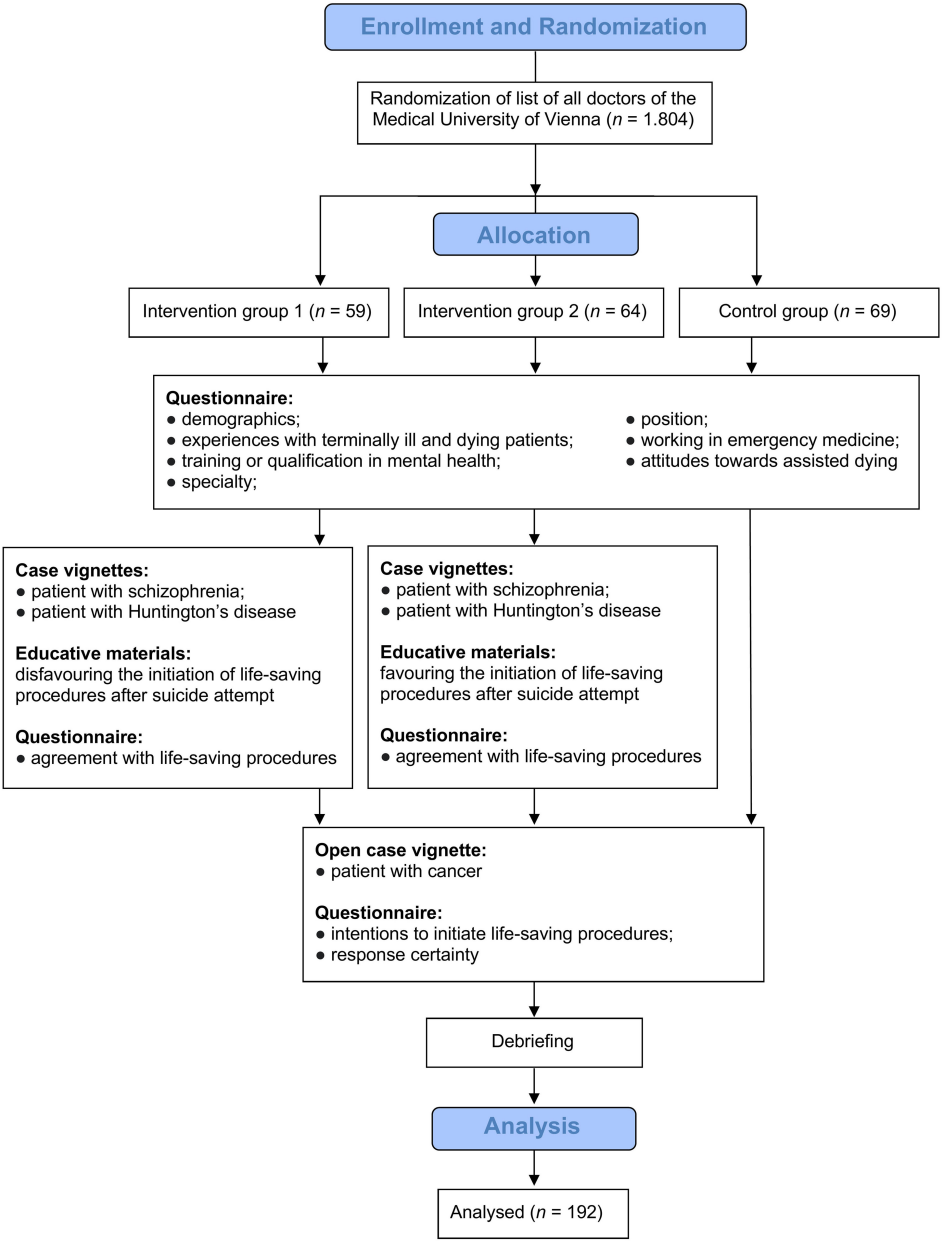
Study flowchart.

Figure 1 shows the study flowchart. Once inclusion criteria were checked and informed consent was provided, participants in all groups were asked to answer a set of socio-demographic questions, including information on position, specialty, and education and training. Further, participants' experiences with terminally ill and dying patients as well as attitudes toward assisted dying were measured.

Individuals in the experimental groups 1 and 2 were then asked to read two case vignettes about patients in emergency situations. Case vignette 1 was about a 43-year-old patient with a 20-year-long history of schizophrenia who intended to end his life by drug overdosing. Case vignette 2 described a 35-year-old patient who intended to die by self-immolation 1 week after being diagnosed with Huntington's disease (leading to severe burns on 80% of his body surface). Both vignettes stated that the patients had raised a wish to die/were suicidal. Study participants were asked to imagine they were seeing these patients. After the presentation of the vignettes, participants in the experimental group 1 were presented with a brief educative text *disfavouring* the initiation of life-saving procedures (meaning to accept the wish to die). Participants in the experimental group 2 were presented with a brief educative text *favouring* the initiation of life-saving procedures (meaning not accepting the wish to die). Participants in both experimental groups were then asked to rate their agreement with the recommended measures for each case study, respectively.

Participants in the control group did not see any of these two case vignettes. All three groups, however, were presented with another case vignette. Note that this was the third vignette for participants in group 1 and 2, but the first (and only) vignette in the control group.

This was an open case vignette about a 72-year-old cancer patient who had intended to end his life with the application of a drug, pentobarbital. Individuals in all study arms were asked to imagine they were seeing this terminally ill patient in the emergency department and were then asked to choose one of the two options: (a) not initiating life-saving measures and accepting the patient's wish to die, or (b) initiating life-saving measures and not accepting the patient's wish to die. After their rating, participants were also asked to rate their response certainty on a 10-point Likert scale.

The case studies presented in the current study were based on actual descriptions of medical emergency situations described by Krones in educative materials (2018). The case vignettes and recommendations were also featured in an educative newsletter that was sent out to all German speaking physicians, including physicians from Austria, subscribing to www.coliquio.de. These materials emphasised the acceptance of death wish in the respective case vignettes, triggering controversies in Austria and Germany particularly. We adapted the recommendations to include a version favouring the initiation of life-saving measures. The brief text was developed in accordance with suicide prevention literature (Sonneck et al., 2016).

At the end of the questionnaire, the purpose of the study was disclosed to participants. Also, all participants were informed that from a suicide-preventive point of view, the recommendations favouring non-initiation of life-saving measures without any additional in-depth discussion about the individual cases were problematic mainly because a proper assessment of the wish to die, as suggested in the presented information texts, was often impossible without additional information and sheer speculation. Further, valuable time would be lost if cases would be discussed in detail regarding intent in the emergency setting before initiating life-saving measures.

### Outcome Measures

#### Agreement With Life-Saving Measures in Case Vignettes About Different Patients' Scenarios

After reading the case vignettes about a patient with schizophrenia and about a patient with Huntington's disease, participants in the two intervention groups were asked to rate their agreement with the measures recommended by the educative materials (i.e., intervention group 1: disfavouring the initiation of life-saving procedures, meaning to accept the wish to die; intervention group 2: favouring the initiation of life-saving procedures, meaning not accepting the wish to die) for each case vignette, respectively (i.e., case vignette 1: patient with schizophrenia; case vignette 2: patient with Huntington's disease) on a 10-point Likert scale from 1 (strongly disagree) to 10 (strongly agree). To create an outcome variable for agreement with life-saving measures, we reverse coded participants' responses in intervention group 1 resulting in a 10-point Likert scale from 1 (agreement with wish to die) to 10 (agreement with life-saving measures) for participants in both intervention groups.

#### Primary Outcome: Intentions to Initiate Life-Saving Procedures in Open Case Vignette

The primary outcome in the presented study were intentions to initiate life-saving measures in the open case vignette about a 72-year-old cancer patient. Participants were asked to either (a) accept the patient's wish to die, or (b) not accept the patient's wish to die and initiate life-saving measures.

#### Response Certainty of Answer in Open Case Vignette

Individuals rated their response certainty of the primary outcome (i.e., the decision in the open case vignette) on a 10-point Likert scale from 1 (very uncertain) to 10 (very certain).

#### Attitudes Toward Assisted Dying

Attitudes toward assisted dying were measured with 4 questions that were used in the British Social Attitudes survey of public opinions (Clery et al., 2007). Respondents rated their level of agreement with 4 statements about assisted dying practises, including euthanasia and physician-assisted suicide (e.g., “First, a person with an incurable and a painful illness, from which they will die—e.g., someone dying of cancer. Do you think that, if they ask for it, a doctor should ever be allowed by law to end their life, or not?”), on a 4-point Likert scale from 1 (definitely should not be allowed) to 4 (definitely should be allowed). This questionnaire was before used in studies on physicians' attitudes to euthanasia and physician-assisted suicides (Seale, 2009). In accordance with Seale (2009) mean scores across all items were calculated to create an Assisted Dying Attitudes (ADAtt) total score ranging from 1 (low support for assisted dying) to 4 (high support for assisted dying) (Cronbach's α = 0.91).

#### Personal Experiences With Terminally Ill and Dying Patients

Based on Leisch (2019), experiences in working with terminally ill and dying patients was assessed by asking participants about their agreement with the four statements: “I have experiences in assisting in caring for a terminally ill individual”, “I have experiences in caring for a terminally ill individual on my own”, “I have experiences in medically treating terminally ill individuals”, and “I have experiences in hospice care”. Based on participants' answer, we created the dichotomous variable “experience” vs. “no experience”.

#### Position, Specialty, and Education and Training

Participants were asked about their position (“intern/resident” or “attending”), specialty (“anaesthesia/intensive care”, “surgical disciplines”, “internistic disciplines”, “neurology, psychiatry, and psychotherapeutic medicine”, or “others”), whether they worked in emergency medicine (yes vs. no), and about extra training or qualification in mental health or psychology (yes vs. no).

### Power Analysis

A power analysis using G^*^Power 3.1.9.2 (Faul et al., 2007) indicated that an χ^2^ test to analyze differences among three groups (i.e., intervention group 1, intervention group 2, and control group; *df* = 2) with a dichotomous outcome (i.e., decision for the initiation of life-saving procedures vs. decision for the initiation of life-saving procedures), using an alpha-level of 0.05 and a power of 0.80 would require a total sample size of 155 participants to detect an effect size w of 0.25.

### Data Analysis

A binary logistic regression analysis was used to investigate whether participants in the three different study groups (i.e., intervention group 1, intervention group 2, and control group) differed in terms of their intentions to initiate life-saving procedures in the open case vignette (primary outcome).

To test for effects of experiences with terminally ill and dying patients, extra training or qualification in mental health or psychology, specialty, position, whether doctors worked in emergency medicine, sociodemographic variables (i.e., gender and age), and attitudes toward assisted dying on intentions to initiate life-saving procedures in the open case vignette, we calculated separate binary logistic regression analyses. Subsequently, all of the above variables were used simultaneously in an adjusted multivariable model.

We calculated a Kruskal-Wallis test to analyze differences in participants' response certainty of their decision in the open case vignette.

To test for differences in participants' agreement with life-saving measures in the case vignettes about a patient with schizophrenia and about a patient with Huntington's disease among the two experimental groups, Mann-Whitley U-tests were used.

Further, differences in the agreement with life-saving measures in the presented recommendation among the two case vignettes (case 1: patient with schizophrenia vs. case 2: patient with Huntington's disease) were analysed with a Wilcoxon signed-rank test.

### Ethics Statement

The study was approved by the Ethics Committee of the Medical University of Vienna and the Vienna General Hospital (study protocol 2233/2018). Participants provided consent by clicking on “Continue” and starting the online survey. The trial was registered with the German Clinical Trials Register (DRKS00024953, 31 March 2021, www.drks.de).

## Results

### Study Participant Characteristics

In total, 192 doctors from the Medical University participated in the study (intervention group 1 with educative materials disfavouring the initiation of life-saving procedures: *n* = 59; intervention group 2 with educative materials favouring the initiation of life-saving procedures: *n* = 64; controls: *n* = 69). *N* = 90 participants were female (46.88%) and the mean age was 41.80 years (*SD* = 11.30), ranging from 25 up to 66 years. Characteristics of participants were similar between groups as indicated by χ^2^ and Eta coefficient tests (Table 1).

**Table 1:** Descriptive statistics for intervention group #1: reading educative material disfavouring life-saving procedures (*n* = 59), intervention group #2: reading educative material favouring life-saving procedures (*n* = 64), and control group: no educative material (*n* = 69).

**Variable**	**Intervention group #1**	**Intervention group #2**	**Control group**	**χ^**2**^*/*η**
Age *M* (*SD*)	42.81 (12.36)	41.69 (10.71)	41 (10.93)	0.07^a^
Gender
Females *n* (%)	28 (48.28)	25 (39.06)	37 (54.41)	3.14^b^
Males *n* (%)	30 (51.72%)	39 (60.94)	31 (45.59)	
Position
Intern/resident *n* (%)	18 (30.51)	23 (35.94)	25 (37.31)	0.70^b^
Attending *n* (%)	41 (69.49)	41 (64.06)	42 (62.69)	
Specialty
Anaesthesia/intensive care *n* (%)	11 (18.64)	11 (17.19)	13 (18.84)	7.79^c^
Surgical disciplines *n* (%)	11 (18.64)	17 (26.56)	16 (23.19)	
Internistic disciplines *n* (%)	10 (16.95)	11 (17.19)	13 (18.84)	
Neurology, psychiatry, and psychotherapeutic medicine *n* (%)	7 (11.86)	7 (10.94)	7 (10.14)	
Others *n* (%)	13 (22.03)	16 (25.00)	19 (27.54)	
More than one specialty *n* (%)	7 (11.86)	2 (3.13)	1 (1.45)	
Doctors working in emergency medicine *n* (%)	24 (40.68)	21 (32.81)	22 (31.88)	1.27^b^
Extra training or qualification in mental health or psychology *n* (%)	10 (16.95)	16 (25.00)	17 (24.64)	1.46^b^
Experiences with terminally ill and dying patients *n* (%)	53 (89.83)	59 (92.19)	63 (91.30)	0.29^b^
Pro-life-saving procedures in open case vignette *M* (*SD*)	20 (37.04)	25 (39.68)	30 (45.45)	0.94^b^
Response certainty of answer in open case vignette *M* (*SD*)	6.93 (2.72)	7.08 (2.40)	7.52 (2.60)	0.10^a^
Attitudes toward assisted dying *M* (*SD*)	2.06 (0.91)	2.33 (0.82)	2.25 (0.99)	0.12^a^

*Frequencies (n), percentages (%), means (M), and standard deviations (SD) provided for each group, as well as χ^2^ values from χ^2^ tests and Eta coefficients from Eta coefficient tests testing group differences.*

a *Eta coefficient*.

b *χ^2^ test result. df = 1*.

c *χ^2^ test result. df = 5*.

### Primary Outcome: Intentions to Initiate Life-Saving Procedures in Open Case Vignette

Overall, the majority of participants (*n* = 108, 56.25%) reported to accept the patients' wish to die and decided against the initiation of life-saving procedures. Participants in the different study arms did not differ in their intentions to initiate life-saving measures, χ^2^(2) = 0.94, *p* = 0.63. Further, no effects of experiences with terminally ill and dying patients [χ^2^(1) = 1.09, *p* = 0.30], training and qualification in mental health [χ^2^(1) = 0.62, *p* = 0.43], specialty [χ^2^(5) = 1.90, *p* = 0.89], position [χ^2^(2) = 0.73, *p* = 0.70], whether doctors worked in emergency medicine [χ^2^(1) = 0.01, *p* = 0.91], gender [χ^2^(1) = 1.45, *p* = 0.23], and age [χ^2^(1) = 0.07, *p* = 0.79] were found. Attitudes toward assisted dying predicted intentions to initiate life-saving procedures, χ^2^(1) = 14.19, < 0.001. Low support of assisted dying was associated with a tendency to decide for life-saving measures.

The adjusted model including all predictors was significant [χ^2^(15) = 37.82, *df* = 15, *p* < 0.001]. Attending position, male gender, low age, and low support for assisted dying predicted a decision for life-saving measures (Table 2).

**Table 2:** Logistic regression analysis for predictors for intentions to initiate life-saving procedures in open case vignette.

	**Crude**					**Adjusted**				
	**B**	**SE**	**df**	**OR (95% CI)**	***p***	**B**	**SE**	**df**	**OR (95% CI)**	***p***
Group (Ref: control group)			2		0.63			2		0.15
Intervention group 1 (pro dying)	−0.35	0.37	1	0.71 (0.34, 1.47)	0.35	−0.88	0.46	1	0.41 (0.17, 1.03)	0.06
Intervention Group 2 (pro living)	−0.24	0.36	1	0.79 (0.39, 1.59)	0.51	−0.54	0.42	1	0.58 (0.26, 1.33)	0.20
Experiences with terminally ill and dying patients										
(Ref: no experience)										
With experience	−0.53	0.51	1	0.59 (0.22, 1.60)	0.30	−1.20	0.63	1	0.30 (0.09, 1.02)	0.05
Extra training in mental health (Ref: no training)										
With training	0.28			1.33 (0.66, 2.67)	0.43	−0.16	0.48	1	0.85 (0.33, 2.17)	0.73
Specialty (Ref: anaesthesia/intensive care)			5		0.89			5		0.79
Surgical disciplines	−0.02	0.48	1	0.98 (0.39, 2.52)	0.97	0.49	0.59	1	1.63 (0.51, 5.17)	0.41
Internistic disciplines	0.19	0.50	1	1.22 (0.46, 3.21)	0.70	0.22	0.59	1	1.24 (0.39, 3.97)	0.71
Neurology, psychiatry, and psychotherapeutic medicine	0.23	0.57	1	1.26 (0.41, 3.87)	0.69	0.84	0.76	1	2.32 (0.52, 10.31)	0.27
Others	0.17	0.46	1	1.18 (0.48, 2.94)	0.72	0.73	0.59	1	2.07 (0.65, 6.63)	0.22
More	−0.82	0.88	1	0.44 (0.08, 2.45)	0.35	0.02	0.96	1	1.02 (0.15, 6.75)	0.98
Position (Ref: intern/resident)			2		0.70			2		**0.02**
Attending	0.26	0.32	1	1.29 (0.69, 2.41)	0.42	1.57	0.56	1	4.80 (1.59, 14.54)	** <0.01**
Emergency medicine (Ref: no)										
In emergency medicine	0.04	0.31	1	1.04 (0.56, 1.92)	0.91	0.21	0.39	1	1.23 (0.57, 2.64)	0.59
Gender (Ref: male)										
Female	−0.37	0.30	1	0.69 (0.38, 1.26)	0.23	−0.86	0.38	1	0.42 (0.20, 0.89)	**0.02**
Age	−0.004	0.01	1	1.00 (0.97, 1.02)	0.79	−0.06	0.03	1	0.94 (0.89, 0.99)	**0.01**
Attitudes toward assisted dying (ADAtt)	−0.68	0.19	1	0.51 (0.35, 0.74)	** <0.001**	−1.10	0.24	1	0.33 (0.21, 0.53)	** <0.001**
Constant						5.16	1.38	1	174.74	** <0.001**
Omnibus-tests						*χ^2^* = 37.82, *df* = 15, *p*<**0.001**				
Nagelkerkes *R^2^*						0.26				
Hosmer and Lemeshow						*χ^2^* = 8.30, *df* = 8, *p* = **0.41**				

*Ref, Reference group; OR, Odds Ratio, CI, Confidence Interval. Significant P-values are in bold*.

### Secondary Outcomes

No significant differences in response certainty of the decision about the open case vignette among the three study groups were found, χ^2^(2) = 2.41, *p* = 0.30.

Agreement with life-saving measures were not significantly different among the two experimental groups in the case vignette about a patient with schizophrenia (*U* = 1649.50, *p* = 0.43) and in the case vignette about a patient with Huntington's disease (*U* = 1581.50, *p* = 0.25).

Participants reported significantly higher agreement with life-saving measures in the case vignette about a patient with schizophrenia (*Mdn* = 9.00) than in the case vignette about a patient with Huntington's disease (*Mdn* = 8.00), *z* = −4.73, *p* < 0.001, *N* = 120, *r* = 0.43.

## Discussion

This study is the first that assessed immediate effects of educative materials that either support non-initiation of life-saving measures after a suicide attempt or recommend life-saving measures on intentions to initiate such measures in physicians in a randomised controlled trial. The materials used were constructed based on original opinionated educative materials targeting physicians, using the same case vignettes.

We found no immediate effect of educative material on doctors' intentions to initiate life-saving procedures after a patient's suicide attempt. Further, personal experiences with terminally ill and dying patients and extra training in mental health did not appear to have an effect on those intentions. Also, decision patterns were independent from whether doctors worked in emergency medicine and did not differ among different medical specialties.

Rather, the present findings indicate that intentions to initiate life-saving measures after a patient's suicide attempt were largely depending on personal attitudes toward assisted dying. Doctors with pro-assisted dying attitudes showed stronger disagreement with life-saving measures and had a more accepting opinion toward the patients' wish to die, regardless of what educative materials they were presented with. Other variables at least partially explaining differences in intentions to initiate life-saving measures included doctors' position, gender, and age. Stronger attitudes in favour of life-saving were seen among more senior physicians, males, and younger doctors. Also the specific patient case and scenario, i.e., his or her diagnosis (and prognosis), appeared to make some difference. Specifically, attitudes in favour of life-saving were higher in a patient with a severe psychiatric disorder (schizophrenia) as compared to a terminally ill patient (Huntington's disease with severe burns).

Some of the present findings are consistent with previous studies. Consistent with findings from a French (Peretti-Watel et al., 2005) and a British study (Seale, 2009), younger physicians showed more attitudes in favour of life-saving. In line with Abohaimed et al. (2019), more senior physicians showed more favourable opinions toward life-saving procedures. This may be attributed to the fact that more senior physicians may have treated more patients and may be more aware of treatment alternatives. Our finding that physicians were more accepting of non-life-saving treatment in terminally ill patients (as compared to a psychiatric patient) is also consistent with previous research (Fried et al., 1993; Ryynänen et al., 2002).

The present study indicated some differences to previously published literature, where attitudes and opinions about life-saving treatment varied with medical specialty (Peretti-Watel et al., 2005; Seale, 2009) and with experiences with terminally ill and dying patients (Grassi et al., 1999; Peretti-Watel et al., 2005; Abohaimed et al., 2019). Further, previous findings (Seale, 2009; Abohaimed et al., 2019) suggested attitudes more positive of euthanasia in males whereas we found an opposite pattern with higher intentions to initiate life-saving measures in males. Considering that previous studies have not typically used actual case vignettes and did not assess intentions to initiate life-saving measures (but rather assessed general opinions on euthanasia and assisted dying), findings of this study might not be fully comparable. Further, this study was conducted at a major university hospital and findings in this setting might not be representative of smaller or less specialised clinical settings.

### Limitations

The study has some limitations. First, we measured effects of educative material after a brief one-time exposure to educative materials only. It remains unclear whether repeated exposure might have a stronger effect. In the light of ongoing discussions on end-of-life decisions, assisted suicide, patient will, and euthanasia, frequent exposure to educative materials as well as discussion items in media are very likely and might still have a relevant impact. Second, we assed data on a fairly mixed group of physicians from one large university hospital, with some underrepresented specialties. Further, we cannot rule out selection bias due to interest in the study topic. A comparison of baseline data suggests that physicians assigned to the three study groups did not differ on important baseline characteristics, suggesting that the randomisation process worked regarding these variables, and should not result in biassed assessments in the effect of the educative materials. The present findings on the effects of other variables (beyond media impact) such as attitudes, however, might not be representative of the entire staff or beyond the university hospital. Further, there are other variables beyond those assessed that might have an impact on decision-making. Specifically, we did not ask about religious beliefs which may also attitudes toward end-of-life care (Ryynänen et al., 2002; Seale, 2009; Abohaimed et al., 2019). Further, outcome variables were assessed post-exposure to the material only. But a successful randomisation process generally compensates for a lack of baseline characteristics. Finally, this study measured attitudes only and did not capture actual behaviour (i.e., actual decisions in emergency situations).

### Implications and Recommendation

With the public and professional debate over end-of-life decisions lately becoming more active (Emanuel, 1994; Moskop and Iserson, 2001; Emanuel et al., 2016; Goligher et al., 2017), and a number of countries adopting increasingly liberal legislation, including Austria, where assisted suicide will be legalised as with 2022 (https://www.vfgh.gv.at/downloads/VfGH-Erkenntnis_G_139_2019_vom_11.12.2020.pdf), discussions regarding ethical aspects of euthanasia and assisted suicide and how to respond to patients with death wished are highly controversial (Materstvedt et al., 2003; Sontheimer, 2008; Olié and Courtet, 2016; Henman, 2017). Considering these active, and often highly opinionated, debates and the timeliness of the topic area, stronger guidance appears highly warranted in order to facilitate a state-of-the art decision-making on how to manage such instances of suicide attempts. Even though no effect of one-time exposure to opinionated material either favouring or disfavouring physicians' intentions to initiate life-saving procedures was found in the present study, we cannot rule out that repetitive exposure to opinionated material circulating in public media might still have an impact on attitudes, and potentially, decision-making. This is concerning if the discussion lacks sufficient depth in order to properly address patient needs. We were unable to assess if the opinionated materials presented in this study resulted in any useful discussions about the topic. No matter which specific decision would be deemed most accurate in a specific patient case, any helpful discussion would always carefully reflect about the issue, and aim for an understanding of the individual patient case that is as complete as possible. In the current case vignettes, which were drawn from a real source, much of the relevant information was not provided or discussed. Any effect of these vignettes to increase pro-end-of-life considerations would have been highly concerning.

Particularly in times of legislation reforms with ongoing public debates on end-of-life decisions, further insights about mechanisms underlying doctors' attitudes regarding these decisions and the role of media material in influencing opinions are highly warranted. There is a strong need for good-quality in-depth discussions regarding end-of-life decisions among medical professionals and the general public and to develop well-founded and non-opinionated guidelines.

## Conclusion

One-time exposure to educative materials related to end-of-life-decisions does not appear to influence intentions to provide life-saving support after a suicide attempt in doctors at a university clinic. Intentions to initiate life-saving procedures after suicide attempts appear mainly influenced by personal opinions on the topic but also by the specific patient case. Education and discussion efforts in this area need to acknowledge the important roles of personal opinions and attitudes for decision-making processes in this topic area and should aim for providing accurate well-reflected inter-disciplinary professional guidance about how to manage such situations.

## Data Availability Statement

The datasets presented in this article are not readily available because of a non-distribution agreement with the data protection commission of the Medical University of Vienna. Requests to access the datasets should be directed to Thomas Niederkrotenthaler, thomas.niederkrotenthaler@meduniwien.ac.at.

## Ethics Statement

The study was approved by the Ethics Committee of the Medical University of Vienna and the Vienna General Hospital (study protocol 2233/2018). Participants provided consent by clicking on “Continue” and starting the online survey.

## Author Contributions

MB and TN contributed to conception and design of the study, and conducted the study. MB performed the statistical analysis, supervised by TN. MB wrote the first draft of the manuscript. TN wrote sections of the manuscript. Both authors contributed to manuscript revision, read, and approved the submitted version.

## Conflict of Interest

The authors declare that the research was conducted in the absence of any commercial or financial relationships that could be construed as a potential conflict of interest.

## Publisher's Note

All claims expressed in this article are solely those of the authors and do not necessarily represent those of their affiliated organizations, or those of the publisher, the editors and the reviewers. Any product that may be evaluated in this article, or claim that may be made by its manufacturer, is not guaranteed or endorsed by the publisher.
